# Risk assessment and prediction model of renal damage in childhood immunoglobulin A vasculitis

**DOI:** 10.3389/fped.2022.967249

**Published:** 2022-08-17

**Authors:** Ruqian Fu, Manqiong Yang, Zhihui Li, Zhijuan Kang, Mai Xun, Ying Wang, Manzhi Wang, Xiangyun Wang

**Affiliations:** ^1^Academy of Pediatrics of University of South China, Changsha, China; ^2^Department of Nephrology and Rheumatology of Hunan Children's Hospital, Changsha, China; ^3^Department of Pediatrics, Hunan Provincial People's Hospital, The First Affiliated Hospital of Hunan Normal University, Changsha, China; ^4^Department of Pediatrics of Changsha Central Hospital, Changsha, China; ^5^Department of Pediatrics of Changsha First People's Hospital, Changsha, China

**Keywords:** children, immunoglobulin vasculitis, renal damage, clinical predictive model, nomogram

## Abstract

**Objectives:**

To explore the risk factors for renal damage in childhood immunoglobulin A vasculitis (IgAV) within 6 months and construct a clinical model for individual risk prediction.

**Methods:**

We retrospectively analyzed the clinical data of 1,007 children in our hospital and 287 children in other hospitals who were diagnosed with IgAV. Approximately 70% of the cases in our hospital were randomly selected using statistical product service soltions (SPSS) software for modeling. The remaining 30% of the cases were selected for internal verification, and the other hospital's cases were reviewed for external verification. A clinical prediction model for renal damage in children with IgAV was constructed by analyzing the modeling data through single-factor and multiple-factor logistic regression analyses. Then, we assessed and verified the degree of discrimination, calibration and clinical usefulness of the model. Finally, the prediction model was rendered in the form of a nomogram.

**Results:**

Age, persistent cutaneous purpura, erythrocyte distribution width, complement C_3_, immunoglobulin G and triglycerides were independent influencing factors of renal damage in IgAV. Based on these factors, the area under the curve (AUC) for the prediction model was 0.772; the calibration curve did not significantly deviate from the ideal curve; and the clinical decision curve was higher than two extreme lines when the prediction probability was ~15–82%. When the internal and external verification datasets were applied to the prediction model, the AUC was 0.729 and 0.750, respectively, and the Z test was compared with the modeling AUC, *P* > 0.05. The calibration curves fluctuated around the ideal curve, and the clinical decision curve was higher than two extreme lines when the prediction probability was 25~84% and 14~73%, respectively.

**Conclusion:**

The prediction model has a good degree of discrimination, calibration and clinical usefulness. Either the internal or external verification has better clinical efficacy, indicating that the model has repeatability and portability.

**Clinical trial registration:**

www.chictr.org.cn, identifier ChiCTR2000033435.

## Introduction

Immunoglobulin A vasculitis (IgAV), formerly called the Henoch-Schönlein purpura (HSP), is characterized by the deposition of immune complexes on the walls of small vessels and is the most common type III hypersensitivity-mediated vasculitis in children (1). The clinical manifestations of IgAV are nonthrombocytopenic purpura, abdominal pain, gastrointestinal bleeding, arthritis and renal damage. It has been reported that the annual incidence of IgAV is ~3-26.7/100,000 (2), of which 30% to 50% of cases result in renal damage (2–4), that is, IgAV with nephritis (IgAVN). The clinical manifestations of IgAVN are hematuria, proteinuria, nephrotic syndrome and renal dysfunction, which usually occur within 6 months of the course of IgAV (3, 4). Most patients generally have a good prognosis, but repeated illness and a prolonged course of disease can lead to end-stage renal disease, which occurs in ~1 to 10% of children (5, 6), creating huge economic, physical and mental burdens to patients and their families. At present, almost all clinical studies on IgAV focus on the risk factors for IgAVN, and there is still a lack of quantitative tools for individualized risk prediction and benefit assessment of renal damage in children with IgAV. The purpose of this paper was to explore the risk factors for IgAVN, construct a clinical prediction model, and evaluate its clinical efficacy. Finally, the prediction model is shown in the form of a nomogram to provide a scientific quantitative tool for individual risk prediction and assessment of benefits for patients.

## Materials and methods

### Research subjects

Subjects enrolled in the study were hospitalized in Hunan Children's Hospital from January 2018 to December 2018 and in Hunan Provincial People's Hospital, Changsha Central Hospital and Changsha First People's Hospital from January 2018 to December 2019. The patients were clinically diagnosed with IgAV (7) and met the following conditions: ➀ age <18 years; ➁ follow-up time≥6 months; ➂ no history of lupus nephritis, poststreptococcal nephritis, IgA nephropathy, hepatitis-associated glomerulonephritis and other renal diseases; and ➃ no history of rheumatic connective tissue diseases, renal damage, tumors and other diseases. This study was approved by the Ethics Committee of Hunan Children's Hospital (ethical batch number: HCHLL-2020-48) and was registered in the China Clinical Trials Registry (registration number: ChiCTR2000033435).

### Research indicators

Indicators are commonly based on previous literature reports, statistically significant indicators, or indicators considered by the investigator to be clinically relevant. In this study, There are 27 indicators included: sex, age, purpura of external skin of the lower limbs, persistent skin purpura and recurrence, abdominalgia, severe colic, gastrointestinal bleeding, joint involvement, white blood cell count, hemoglobin concentration, platelet count, erythrocyte distribution width, C-reactive protein (CRP), erythrocyte sedimentation rate (ESR), complement C3, complement C4, immunoglobulin G (IgG), IgM, IgA, IgE, triglycerides, total cholesterol, low density lipoprotein, albumin, serum creatinine and uric acid. End point indicator: the occurrence of renal damage in children with IgAV. According to EULAR/PRINTO/PRES criteria for Henoch–Schönlein purpura (7), IgAV with nephritis criteria: Proteinuria > 0.3 g/24 h or > 30 mmol/mg of urine albumin/creatinine ratio on a spot morning sample; Hematuria or red blood cell casts: >5 red blood cells/high power feld or red blood cells casts in the urinary sediment or ≥ 2+ on dipstick.

### Statistical methods

SPSS 18.0 software, Stata 15.0 software and R 3.5.1 language were used to analyze the clinical data. The measurement data with a normal distribution are expressed as the means ± standard deviation (SD), and comparisons between the two groups were performed using the independent sample *t*-test. The measurement data of skewness distribution are expressed as the medians and interquartile ranges (IQR), and the Mann–Whitney *U*-test was used to determine differences between groups. The counting data are expressed as frequencies (percentages), and the chi-square test was used for the analysis of counting data. Univariate analysis and multifactor logistic regression analysis were used to construct a clinical prediction model for the data of the modeling group. The discrimination, calibration and clinical usefulness of the model were evaluated using the AUC, calibration curve and clinical decision curve (DCA). Internal and external verification datasets were used to further verify the clinical effectiveness of the model, which was finally shown in the form of a nomogram. *P* < 0.05 were considered statistically significant.

## Results

### General information

**Figure 1:**
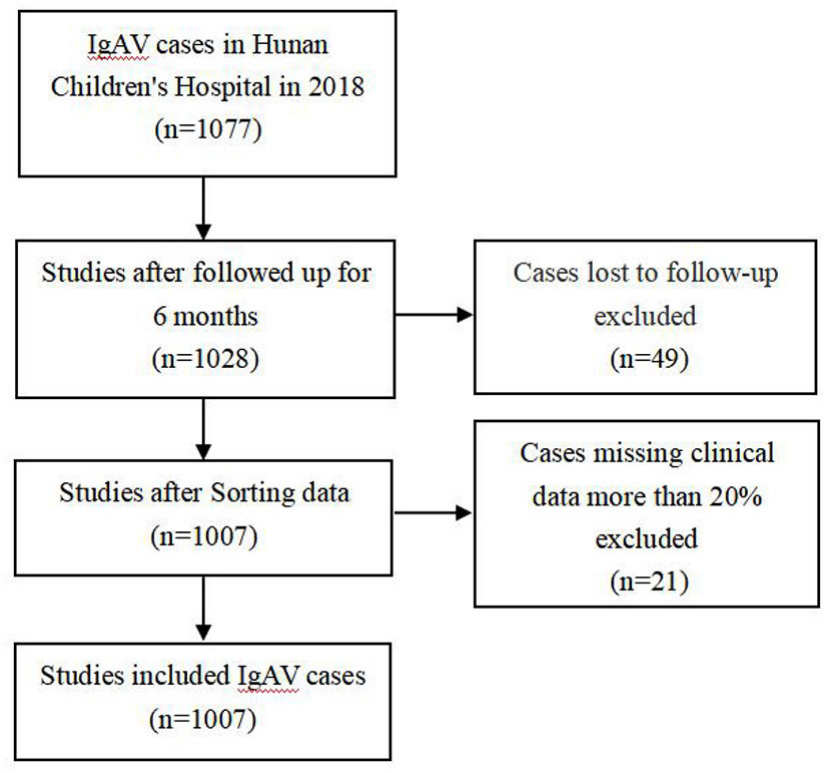
Flowchart of included/excluded cases for studies.

After excluding the cases of loss to follow-up and missing clinical data more than 20% (Figure 1), a total of 1,007 cases were included from Hunan Children's Hospital, including 579 (57.5%) males and 428 (42.5%) females. The median (IQR) age at disease onset was 7.20 (5.64, 9.52) years. A total of 715 cases were randomly selected using SPSS software as a modeling set to establish a clinical prediction model, and the remaining 291 cases were used as the internal verification set. A total of 287 cases, including 155 males (54.0%) and 131 females (46.0%) with a median age of 6.88 (5.16, 9.13) years were included in Hunan Provincial People's Hospital, Changsha Central Hospital and Changsha First People's Hospital as the external verification set. A total of 335 (46.9%) children with IgAV had renal damage. There were 335 (46.9%) cases with IgAVN in the modeling set, 137 cases (46.9%) in the internal verification set and 87 cases (30.3%) in the external verification set.

### Risk factors

Among the 1,007 cases from Hunan Children's Hospital, the occurrence of renal damage within 6 months of IgAV was the dependent variable, and 27 observation indicators of the study subjects were the independent variables. The results of univariate analysis showed that 15 indicators were statistically significant (Table 1). In this study, 11 of the 15 indicators were diagnosed by collinearity, and the results showed that the tolerance of all variables was close to 1 and variance inflation factor (VIF) <10, indicating that there was no collinearity, and the indicators could be included in logistic regression analysis. Fifteen statistically significant indicators from the univariate analysis were included in the multivariate logistic analysis (Table 2); age, persistent cutaneous purpura, erythrocyte distribution width, complement C_3_, immunoglobulin G and triglycerides were independent influencing factors of IgAVN.

**Table 1:** Univariate analysis of observation indicators in 1,007 children with IgAV.

	**IgAVN group (*n* = 472)**	**IgAV group (*n* = 535)**	**Statistical value**	***P***
Age (year)	8.50 ± 2.98	7.04 ± 2.69	8.197	<0.001
Sex (%)			0.111	0.739
Male	274 (58.05)	305 (57.01)		
Female	198 (41.95)	230 (42.99)		
Purpura of external skin of the lower limbs (%)	152 (32.20)	179 (33.46)	0.179	0.672
Persistent skin purpura (%)	155 (32.84)	73 (13.64)	52.746	<0.001
Recurrence (%)	53 (11.23)	24 (4.49)	16.145	<0.001
Abdominalgia (%)	276 (58.47)	299 (55.89)	0.685	0.408
Severe colic (%)	90 (19.07)	95 (17.76)	0.287	0.592
Gastrointestinal bleeding (%)	39 (8.26)	39 (7.29)	0.332	0.564
Joint involvement (%)	303 (64.19)	389 (72.71)	8.459	0.004
White blood cell count (× 10^∧^9/L)	10.33 ± 4.30	10.83 ± 4.33	1.578	0.115
Hemoglobin concentration (g/L)	125.83 ± 12.97	124.46 ± 12.50	−1.701	0.083
Platelet count (× 10^∧^12/L)	334.66 ± 104.49	348.27 ± 95.47	2.159	0.016
Erythrocyte distribution width (%)	13.20 (12.60, 13.90)	12.80 (12.30, 13.40)	6.777	<0.001
CRP (mg/l)	2.61 (1.58, 8.12)	5.60 (2.56, 12.40)	−7.112	<0.001
ESR (mm/h)	9.00 (3.00, 18.00)	11.00 (6.00, 22.00)	−4.262	<0.001
Complement C_3_ (g/L)	0.94 ± 0.19	1.02 ± 0.20	5.944	<0.001
Complement C_4_ (g/L)	0.20 ± 0.07	0.23 ± 0.08	5.468	<0.001
IgG (g/L)	8.95 (6.95, 11.1)	10.10 (8.19, 12.10)	−5.982	<0.001
IgM (g/L)	1.01 (0.78, 1.37)	1.05 (0.82, 1.37)	−1.680	0.093
IgA (g/L)	2.04 (1.54, 2.74)	1.97 (1.51, 2.59)	1.003	0.316
IgE (IU/mL)	56.70 (20.65, 152.00)	68.50 (25.10, 174.00)	−1.377	0.168
Triglycerides (mmol/L)	1.22 (0.93, 1.68)	1.02 (0.75, 1.29)	7.234	<0.001
Total cholesterol (mmol/L)	3.70 (3.22, 4.37)	3.49 (3.12, 3.97)	4.290	<0.001
Low density Lipoprotein (mmol/L)	2.03 (1.65, 2.49)	1.94 (1.60, 2.35)	1.903	0.057
Albumin (g/L)	38.9 (35.90, 41.55)	39.00 (36.80, 41.40)	−1.085	0.278
Serum creatinine (μmol/L)	34.45 (29.00, 41.80)	30.70 (26.40, 36.50)	6.738	<0.001
Uric acid (μmol/L)	249.20 (198.30, 302.00)	228.00 (182.00, 286.00)	3.665	<0.001

*(a) persistent skin purpura: skin purpura lasts for more than 1 month; (b) recurrence: new skin purpura or other systemic complications in patients previously diagnosed with IgAV and asymptomatic for at least 1 month are defined as recurrence; (c) gastrointestinal symptoms: including abdominal pain, severe colic and gastrointestinal bleeding, in which severe colic refers to those who are unable to eat because of diffuse abdominal pain; (d) joint involvement: joint swelling or soft tissue edema around joint pain*.

**Table 2:** Multivariate analysis of 1,007 children with IgAV.

**Variable**	**B**	**Z**	***P***	**OR**	**OR(95%CI)**
Age	0.181	5.79	<0.001	1.198	1.127~1.274
Persistent skin purpura	0.852	4.04	<0.001	2.345	1.551~3.546
Erythrocyte distribution width	0.280	3.99	<0.001	1.323	1.153~1.518
Complement C_3_	−1.551	−3.41	<0.001	0.212	0.087~0.517
Igg	−0.123	−4.10	<0.001	0.884	0.834~0.938
Triglycerides	0.676	4.59	<0.001	1.967	1.473~2.626
Constant	−3.530	−3.22	<0.001	0.029	0.003~0.250

*B, partial regression coefficient; Z, statistics; OR, odds ratio; 95% CI, 95% confidence interval*.

### Establishment of a model

The modeling set establishes the multiple regression equation of the prediction model according to the statistically significant variables and their regression coefficients in the multivariate analysis. P is the probability of occurrence of IgAVN with a value range of 0–1. The closer the *P* value is 1, the greater the probability, and the closer the *P* value is 0, the smaller the probability. A multivariate logistic regression model was established as follows: LogitP = −3.530 + 0.181X_1_ + 0.852X_2_ + 0.280X_3_ −1.551X_4_ −0.123X_5_ + 0.676X_6_.

Note: X_1_: age, X_2_: persistent skin purpura, X_3_: erythrocyte distribution width, X_4_: complement C3, X_5_: immunoglobulin G, X_6_: triglyceride.

### Evaluation and verification of the model

#### Discrimination

**Figure 2:**
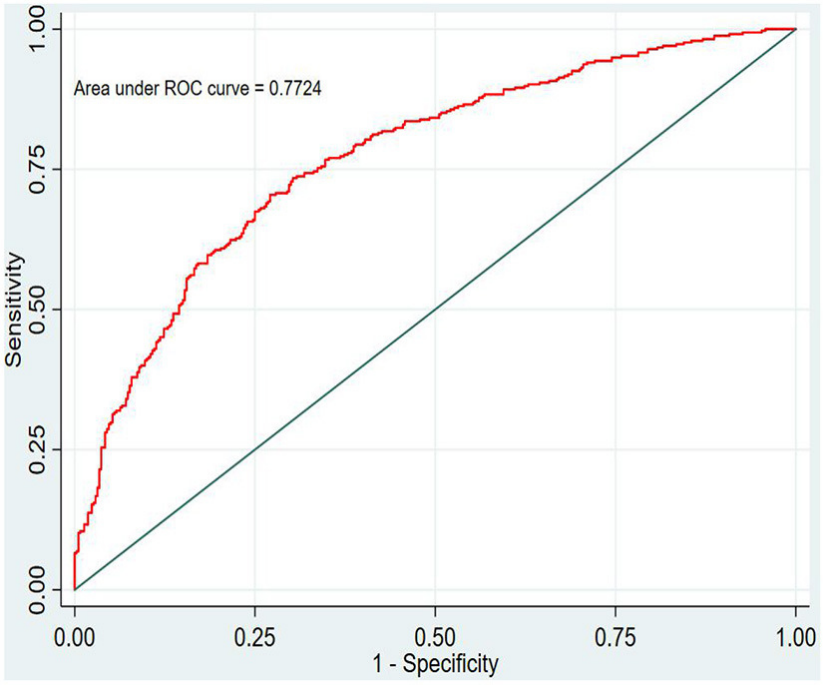
Model set.

**Figure 3:**
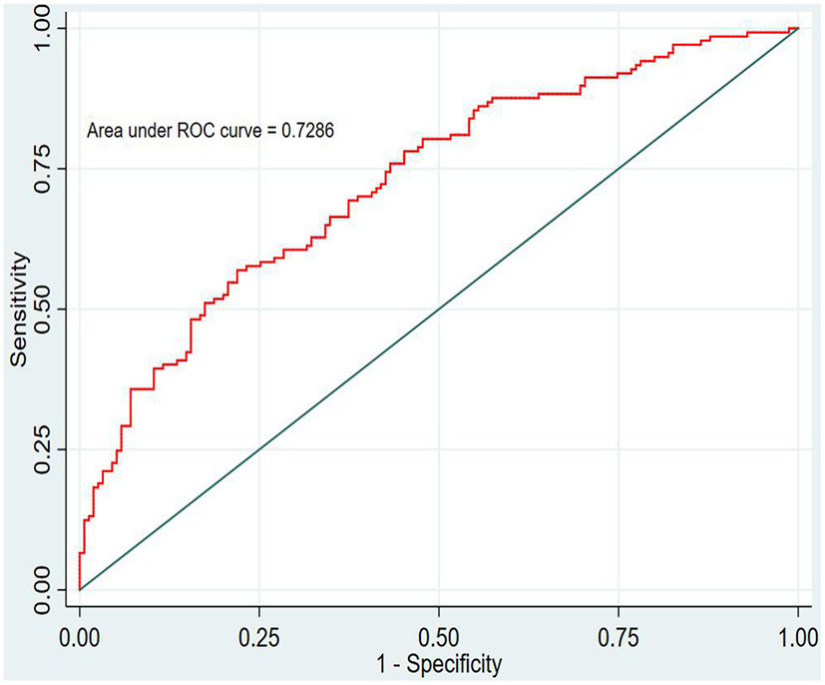
Internal verification set.

**Figure 4:**
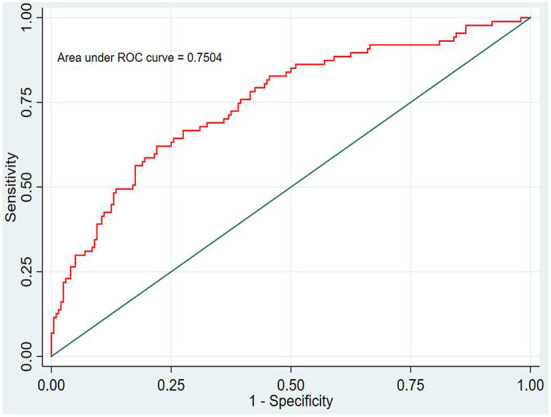
External verification set.

In this study, the prediction probability of IgAVN in the modeling set was expressed by P-m. According to the occurrence of the prediction probability P-m and the actual IgAVN in the modeling set, the ROC curve of the P-m value was constructed using Stata15.0 software (Figure 2), and the discrimination of the IgAVN prediction model was evaluated using the AUC. The ROC curve showed that the AUC of the P-m value in the IgAVN model was 0.772, and the 95% confidence interval was 0.738~0.807. We divided the IgAVN model into the internal verification set and the external verification set, calculated the prediction probability P-in value and P-ex value, respectively, and constructed the ROC curve for these two sets (Figures 3, 4). The AUC of the internal verification set was 0.729, and the 95% CI was 0.671~0.786; the AUC of the external verification set was 0.750, and the 95% CI was 0.688~0.813. There was no significant difference between the model set and two verification sets according to the results of the Z-test used to compare the AUCs.

#### Calibration

**Figure 5:**
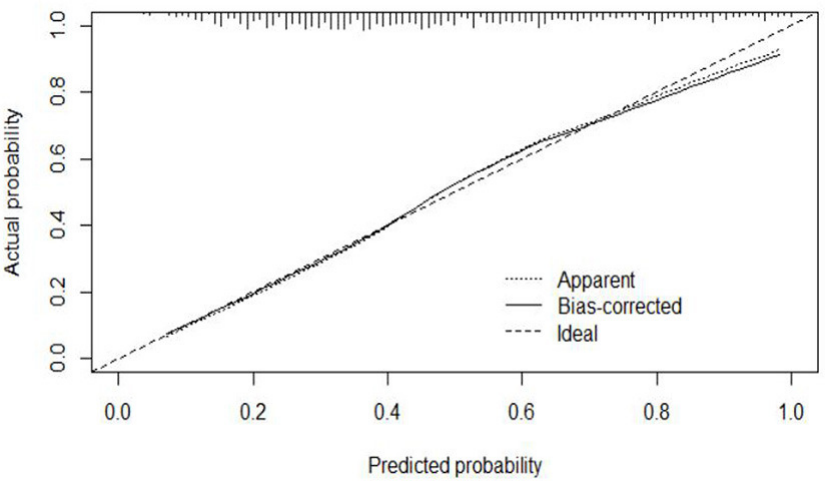
Calibration curve for the model set.

**Figure 6:**
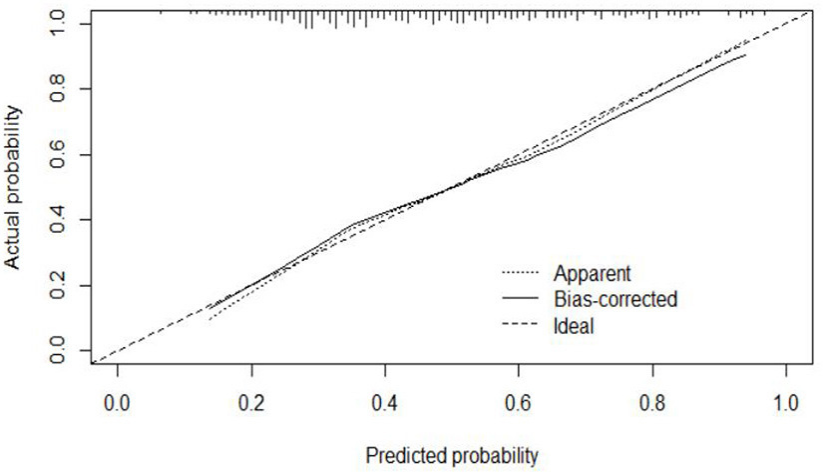
Calibration curve for the internal verification set.

**Figure 7:**
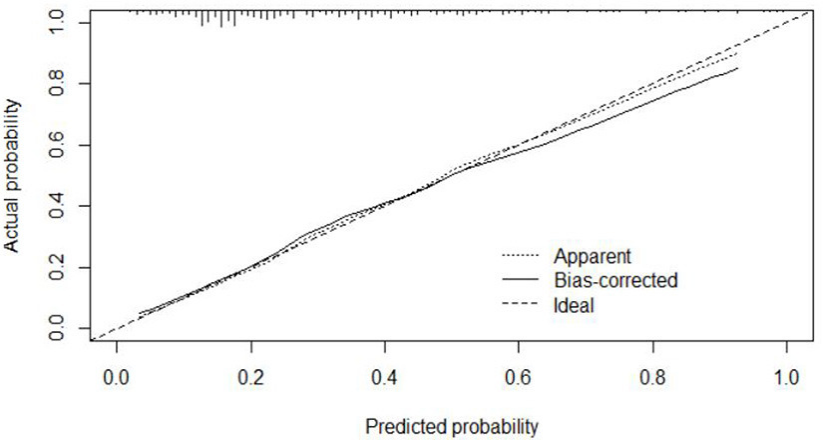
Calibration curve for the external verification set.

The calibration curve of the modeling set was constructed using R3.5.1 software (Figure 5); the x-axis represents the prediction probability of IgAVN, and the y-axis represents the actual probability of IgAVN. The slope of Ideal (diagonal) was 1, which represents the ideal curve; Apparent represents the uncalibrated prediction curve and Bias-corrected represents the calibration prediction curve. The chart shows that the uncalibrated prediction curve and the calibration prediction curve fluctuated around the diagonal and did not significantly deviate from the ideal curve. The calibration curve of the internal verification set (Figure 6) and the external verification set (Figure 7) show that the uncalibrated prediction curve and the calibration prediction curve fluctuated around the diagonal and did not significantly deviate from the ideal curve. The goodness of fit for all three groups was assessed using the Hosmer–Lemeshow test, and the difference was not statistically significant.

#### Clinical usefulness

**Figure 8:**
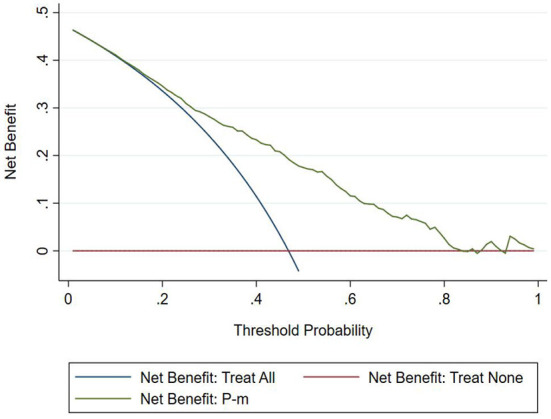
DCA of model set.

**Figure 9:**
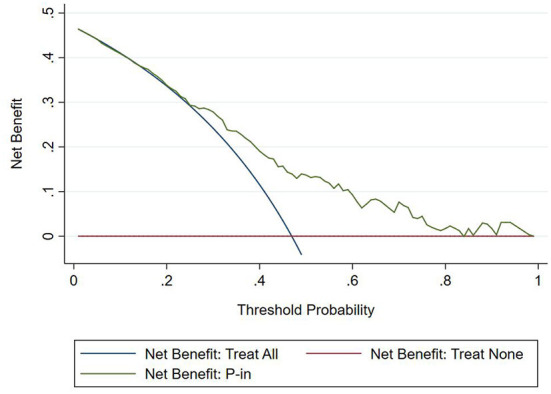
DCA of internal verification set.

**Figure 10:**
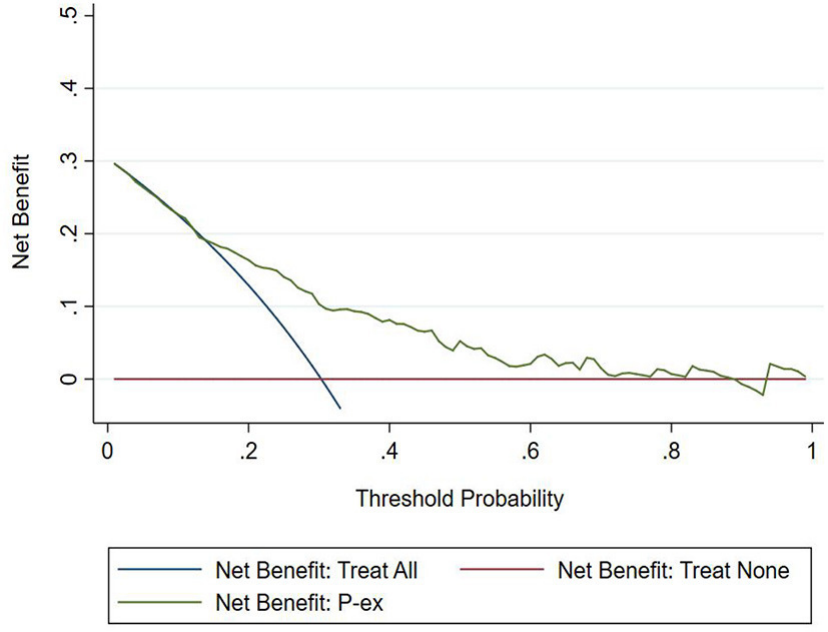
DCA of external verification set.

The DCA chart was constructed using Stata15.0 software (Figure 8). In the DCA diagram, the red horizontal line indicates that when the prediction model in this study lacked renal damage, the clinical net benefit was zero; the blue slash indicates that when all IgAV cases had renal damage, the clinical net benefit was a slope that was negative; and the green curve was based on the curve associated with the IgAVN model in this study. As we can see from Figure 7, when the prediction probability P-m was between 15 and 82%, the green curve was higher than the two extreme lines, and the model can benefit from predicting renal damage in IgAV. The DCA diagram of the internal verification set and the external verification set was constructed according to the IgAV renal damage prediction probability P-in value and P-ex value and the actual IgAV renal damage occurrence situation in these sets (Figures 9, 10). It can be concluded from the two figures that when the predicted probability of P-in was ~25~84% and P-ex was ~14%~73%, the green decision curve was higher than the blue slash line and red cross line in extreme cases, suggesting that children can benefit from the model when predicting renal damage in IgAV within the range of prediction probability.

### Model presentation

**Figure 11:**
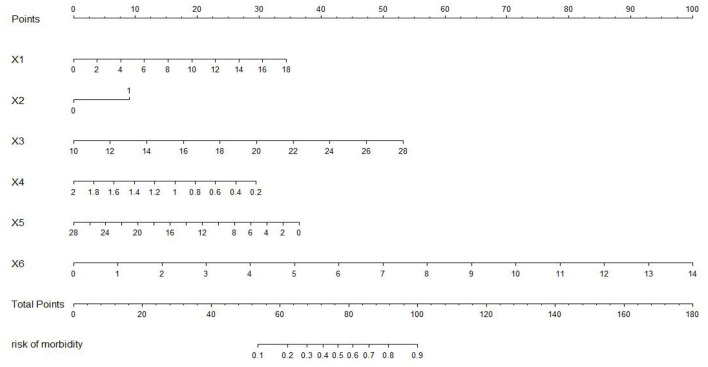
Nomogram of the prediction model. X_1_, age; X_2_, persistent skin purpura; X_3_, erythrocyte distribution width; X_4_, complement C3; X_5_, immunoglobulin G; X_6_, triglyceride.

The nomogram of the IgAVN prediction model was constructed using the rms package of R3.5.1 software (see Figure 11). The nomogram converts the regression equation into an easy-to-understand visual graph, which makes the results of the prediction model more readable and convenient for the risk assessment of patients. In Figure 10, X1–X6 represent the independent variables, and each variable finds the point on the axis corresponding to the line chart and indicates the score of the variable. The total score of each variable adds up to the total score of the corresponding total score scale, and the final total score corresponds to the reading on the risk axis of renal damage, which indicates the probability of renal damage in the child.

## Discussion

IgAV is the most common small vessel inflammatory disease in childhood and worldwide and mainly involves the small vasculature of the skin, joints, gastrointestinal tract and kidneys (8). The clinical manifestations of IgAVN are mainly hematuria and proteinuria, which long-term prognosis depends on the severity of renal damage (9). The influencing factors of IgAVN are complex; a large number of studies have shown that the older the age of the child, the greater the risk of renal damage, which is most likely due to the increase in immune function with age. A systematic review of 13 studies from the year 2000 to 2016 (10) (2,398 children) showed that males older than 10 years of age, gastrointestinal symptoms such as abdominal pain and bleeding, joint involvement, persistent skin purpura, recurrent rash, elevated white blood cells, elevated platelets and decreased complement C_3_ can increase the risk of renal involvement. Some studies have shown that erythrocyte distribution width can be used as a marker of IgAV risk and may be related to the severity of the disease (11). At present, it is controversial whether immunoglobulin G is related to IgAVN. In a retrospective analysis of 250 children with IgAV, elevated cholesterol and low-density lipoprotein was identified as an independent risk factor for IgAVN (12). In this paper, the children with IgAV were followed up for half a year, and renal damage was the end point index. Twenty-seven potential predictive indicators, such as age, clinical manifestations, and serological indices were used in univariate analysis and logistic multivariate analysis The results showed that age, persistent purpura, erythrocyte distribution width, complement C_3_, immunoglobulin G and triglycerides were considered to be independent influencing factors for IgAVN, which is similar to the findings of previous studies on the prediction risk factors for IgAVN.

The construction of a clinical predictive model based on risk factors is a popular and challenging endeavor in current research. A prediction model is a tool to obtain risk or probability by assigning relative weights to each prediction factor to combine multiple prediction factors (13). Clinically, the establishment of a predictive model is one of the important methods used to transform clinical research into clinical application. However, the establishment of a good clinical prediction model is a complex project. The “Transparent Reporting of a multivariable prediction model for Individual Prognosis or Diagnosis” (TRIPOD) (14) published in 2015 has made a relatively complete behavior norm for the establishment of the prediction model, including the construction, evaluation, and verification of the model.

Building a model involves the selection of possible predictors and combining them into a multivariate model, which is usually analyzed by logistic regression or Cox regression. Logistic regression analysis is most commonly used to predict binary events, while Cox regression is often used for long-term prognostic results (15). This paper is a retrospective study, and the content of the study is the risk prediction of renal damage in IgAV. The end point is whether renal damage occurs in IgAV within 6 months of follow-up; thus, it is considered a binary outcome index. Therefore, logistic regression was selected to analyze the predictive factors for IgAVN.

The evaluation model includes the evaluation of discrimination, calibration and clinical usefulness. The degree of discrimination refers to the ability of the prediction model to distinguish between terminal events and nonterminal events; the AUC is generally used to quantify logistic models (14). The closer the AUC is to the value of 1, the better the discrimination of the model, and clinically, the model has a better degree of discrimination when 0.7 < AUC <0.9 (16). The accuracy reflects the degree to which the model correctly estimates the absolute risk; that is, whether the predicted value of the model is consistent with the actual value (16, 17). The positional relationship between the calibration curve and the ideal curve and the Hosmer–Lemeshow test are usually used for evaluation (18). Clinical practicality refers to the clinical net benefit of the patient using the prediction model at a certain threshold probability, which is evaluated by DCA (19). DCA obtains the net benefit value of using the model on this threshold by determining the relationship between the selected predictive probability threshold and the relative values of false-positive and false-negative results (20, 21). The net return is calculated by all possible risk thresholds between the two extremes, that is, zero and maximum risk estimates, representing all negative events and all positive events, respectively (22). If the DCA is higher than two extreme lines, it indicates that the patient can benefit, and the clinical practicability is better (23). In this paper, the AUC of the IgAVN model based on the logistic regression equation was 0.772 (0.738–0.807), suggesting that the renal damage model has a good degree of discrimination. The Hosmer–Lemeshow test of the model, *P* > 0.05, and the calibration curve around the ideal curve in Figure 2 all indicate that the renal damage model has good accuracy. According to the IgAVN model, the DCA is higher than two extreme lines when the value of P-m is ~15~82%, indicating beneficial effects for the child. This shows that the IgAVN model established in this study has a good degree of discrimination, calibration and clinical usefulness. The model is helpful to effectively identify the risk of IgAVN in the clinic and can guide regular revisits and timely treatment of high-risk children with IgAV.

However, the IgAVN model needs to be validated in other populations before it is used in clinical practice. Model verification is not simply repeating the analysis steps established by the model in other individuals to see if they have the same prediction factors and weights but involves application of the new individuals to the established model. According to the prediction factors in the model and the weight assigned (such as the regression coefficient), the predicted value of the new individual is estimated, and the prediction performance of the model is quantified (24–26). The verification of the model includes internal verification and external verification; internal verification reflects the repeatability of the model, and external verification reflects the portability of the model. In this paper, the split sample verification method was used to select the clinical data of children with 70% IgAV in our hospital as the modeling set and the clinical data of children with 30% IgAV as the internal verification set. The clinical data of children with IgAV in other medical centers were used as the external verification set. In these two sets of data, the two types of verification are related to the discrimination, calibration and clinical usefulness of the evaluation model. In the discrimination evaluation, the AUC of the internal verification set was 0.729 (0.671–0.786), and the AUC of the external verification set was 0.750 (0.688–0.813). The AUC of these verification sets was smaller than that of the modeling set, indicating that the discrimination of the model decreased in the smaller dataset. However, Z test results showed no difference in the AUC between the two sets and the modeling set. The results showed that the prediction model still had good discrimination in the verification data. In the evaluation of calibration, the calibration curves for both the internal and external verification sets fluctuated above and below the ideal curve, and the Hosmer–Lemeshow test showed a *P* > 0.05. This shows that the application of the IgAVN model in these two groups of data has good calibration. In the evaluation of clinical usefulness, the DCA curves drawn by the internal and external verification groups were higher than two extreme lines when the P-in value was 25–84% and the P-ex value was 14–73%, respectively, indicating that the prediction model has good clinical usefulness. Therefore, the IgAVN model has good discrimination, calibration and clinical usefulness in both the internal verification group and the external verification group, indicating that the renal damage model shows good repeatability and portability.

In addition, to facilitate the renal damage model in the evaluation of patients, the most commonly used method is to visualize the IgAVN model. At present, the model is usually presented as a nomogram in the research of clinical prediction models. The nomogram has the ability to personalize the prediction, enabling it to identify and assess the risk for each patient (27). In this paper, the nomogram of the IgAVN model is constructed using the rms software package of R statistics software, and the relatively complex regression equation is transformed into a simple visual graph, which is helpful in making effective clinical decisions when the renal damage model is widely used in the clinic. As we can see from the nomogram in this study, in a child with IgAV aged 8 years and 2 months with skin purpura for more than 1 month, red blood cell distribution width 13.6%, complement C_3_ 0.96 g/L, immunoglobulin G 6.92 g/L and triglycerides 1.88 mmol/L, the probability of renal damage is ~82%. It is suggested that the risk of renal damage is high, and clinicians and family members should remain vigilant and have more frequent visits for reexamination if necessary.

In summary, through univariate and multivariate logistic regression analyses, it was concluded that older age, persistent purpura ≥ 1 month, increased erythrocyte distribution width, decreased complement C_3_, decreased immunoglobulin G and elevated triglycerides are independent influencing factors of IgAVN. The IgAVN model based on binary logistic regression has good clinical predictive ability and clinical practicability through the evaluation of clinical efficacy in terms of discrimination, calibration and clinical usefulness. Additionally, internal and external verification of the model revealed good clinical repeatability and portability. The model is presented as a line chart, which provides an effective reference basis for the individual risk assessment of patients, contributes to early warning of potential IgAVN in patients, and maximizes the clinical benefits in children.

## Data availability statement

The raw data supporting the conclusions of this article will be made available by the authors, without undue reservation.

## Ethics statement

The studies involving human participants were reviewed and approved by the Institutional Review Board of the Hunan Children's Hospital.

## Author contributions

RF, ZL, and ZK contributed to conception and design of the study. MY, MX, YW, MW, and XW provided and organized the data. RF wrote the first draft of the manuscript. ZL reviewed sections of the manuscript. All authors contributed to the article and approved the submitted version.

## Funding

This work was supported by Scientific Research Project of Health Commission of Hunan Province in China (202106012359).

## Conflict of interest

The authors declare that the research was conducted in the absence of any commercial or financial relationships that could be construed as a potential conflict of interest.

## Publisher's note

All claims expressed in this article are solely those of the authors and do not necessarily represent those of their affiliated organizations, or those of the publisher, the editors and the reviewers. Any product that may be evaluated in this article, or claim that may be made by its manufacturer, is not guaranteed or endorsed by the publisher.
